# Perioperative predictors of prolonged hospital stay and postoperative nausea and vomiting after metabolic bariatric surgery

**DOI:** 10.1186/s12871-026-04012-6

**Published:** 2026-06-17

**Authors:** Tsung-Yang Lee, Cheng-Hsu Lu, Chiung-Wen Lai, Amina M Illias, Shao-Chun Wu, Yu-Fang Liu, Yung-Fong Tsai

**Affiliations:** 1 Department of Anesthesiology, Jen-Ai Hospital, Taichung, Taiwan; 2 Department of Anesthesiology, Linkou Branch, Chang Gung Memorial Hospital, 5 Fu-Shin Street, Kwei-Shan, Tao-Yuan, Taoyuan, 333 Taiwan; 3 College of Medicine, Chang Gung University, Taoyuan, Taiwan; 4 Department of Anesthesiology, Kaohsiung Branch, Chang Gung Memorial Hospital, Kaohsiung, Taiwan; 5 Department of Anesthesiology, China Medical University Hospital, Taichung, Taiwan

**Keywords:** Anesthesia duration, Metabolic bariatric surgery, Length of stay, Postoperative nausea and vomiting, Predictors

## Abstract

**Background:**

Prolonged length of stay (LOS) and postoperative nausea and vomiting (PONV) are frequent after metabolic bariatric surgery (MBS) and negatively affect recovery. This study aimed to identify predictors of prolonged LOS and PONV in patients undergoing MBS.

**Methods:**

A retrospective cohort of 476 patients undergoing MBS, including laparoscopic adjustable gastric banding (LAGB) and laparoscopic Roux-en-Y gastric bypass (LRYGB), between January 2022 and September 2023 was analyzed. Patient characteristics and perioperative variables were reviewed. Prolonged hospitalization was defined as LOS > 3 days. Multivariable logistic regression modeling was used to identify independent predictors of prolonged LOS.

**Results:**

Among 476 patients, the median LOS was 3 days, and 23.1% required hospitalization > 3 days. Multivariable analysis identified longer anesthesia duration (odds ratio [OR]: 3.05, 95% confidence interval [CI]: 1.70–5.50, *p* < 0.001), greater intraoperative fluid rate (OR: 1.41, 95% CI: 1.11–1.78, *p* = 0.005), asthma (OR: 2.99, 95% CI: 1.09–8.18, *p* = 0.033), and PONV (OR: 2.09, 95% CI: 1.29–3.40, *p* = 0.003) as independent predictors of prolonged hospitalization. These associations remained significant after adjustment for baseline covariates. The type of surgery (LRYGB vs. LAGB) was not an independent predictor of prolonged LOS in the multivariable model (OR: 1.65, 95% CI 0.94–2.87, *p* = 0.079). PONV occurred in 45.6% of patients and was more common in females and younger patients (*p* < 0.001).

**Conclusion:**

Prolonged anesthesia duration, greater intraoperative fluid rate, asthma, and PONV independently predicted extended hospital stay after MBS. Optimizing anesthesia time, fluid therapy, procedure-specific management, and respiratory management may enhance recovery and shorten hospitalization.

**Supplementary Information:**

The online version contains supplementary material available at 10.1186/s12871-026-04012-6.

## Background

The global prevalence of obesity has reached epidemic proportions, and poses substantial health and socioeconomic challenges worldwide [1]. In 2019, obesity attributable to high body mass index (BMI) or excess body weight accounted for an estimated 5.0 million deaths and 160 million disability-adjusted life years worldwide [2]. Projections suggest that global obesity prevalence could approach one-fifth of adults by 2030 [3]. Metabolic bariatric surgery (MBS) is widely regarded as one of the most effective and durable interventions for the management of severe obesity and its associated medical problems [4, 5]. Compared with nonsurgical treatment, substantial evidence indicates that metabolic bariatric procedures yield significant and sustained weight loss, as well as long-term improvements in overall survival [6, 7]. Additionally, postoperative patients often experience a reduced need for obesity-related medications, reflecting improvements in metabolic health [8]. Each 1% increase in surgical utilization could yield millions of additional life-years globally [7].

Procedures such as laparoscopic adjustable gastric banding (LAGB) and laparoscopic Roux-en-Y gastric bypass (LRYGB) have been refined over time with a growing focus on procedural safety and standardization [9, 10]. Nevertheless, the perioperative course in bariatric patients remains challenging, and is influenced by obesity-associated physiological changes and the presence of associated medical problems [11]. In particular, older age has been associated with increased postoperative complication rates in MBS [11]. As surgical safety and efficiency have improved, optimizing postoperative recovery, including minimizing hospital stay and perioperative complications, has become a central focus of modern bariatric care.

Prolonged length of hospital stay after MBS is clinically important, as it is associated with delayed recovery, higher health care costs, and greater risk of postoperative complications or readmissions [12]. Reported predictors of prolonged length of stay (LOS) after MBS include patient demographics, associated medical problems, anesthesia duration, intraoperative fluid balance, and postoperative complications [12–14]. For example, liberal perioperative fluid strategies have been associated with longer hospital stay in surgical patients [15], and longer surgical duration has been identified as a risk factor for delayed postoperative recovery in bariatric anesthesia settings [16]. These variations in results may also stem from the methodological heterogeneity in the included procedures, and reveal the importance of analyzing the impact of specific surgical techniques. Large-scale database analyses have shown that advanced age, higher BMI or burden of associated medical problems, and prolonged operative or anesthesia time are independent predictors of extended LOS after MBS [12, 13]. However, discrepancies among studies persist, likely due to institutional differences in discharge criteria, perioperative pathways, and variable definitions of ‘prolonged’ LOS. In addition, obesity-related physiologic and pharmacokinetic alterations can modify responses to anesthetic and analgesic drugs, potentially influencing postoperative recovery trajectories [17].

Postoperative nausea and vomiting (PONV) is frequent after MBS, with prior reports describing rates as high as ~ 80% in selected cohorts. PONV is associated with decreased satisfaction, prolonged hospital stays, and higher costs [18]. Multimodal antiemetic strategies in fast-track pathways can reduce PONV and are linked to shorter hospitalization [19]. Multiple risk factors, such as female sex, younger age, volatile anesthetics, and postoperative opioids, are well-established determinants of PONV, and multimodal antiemetic with opioid-sparing strategies are recommended [20]. In bariatric populations, emerging evidence (e.g., sugammadex vs. neostigmine) shows PONV reduction, but procedure-specific data remain comparatively limited [21]. The unique physiological and pharmacologic challenges in severe obesity, including increased adipose distribution volume, altered cardiac output, and metabolic changes, can modify drug pharmacokinetic/pharmacodynamic and may attenuate responses to standard antiemetic regimens [17, 22].

Recent studies in bariatric anesthesia show that opioid-free techniques can reduce PONV, while evidence on individualized fluid therapy largely stems from non-bariatric surgical populations and focuses on recovery metrics rather than hard endpoints [15, 23, 24]. Despite advances, few studies have concurrently evaluated PONV and prolonged LOS in the same bariatric cohort. Understanding how factors such as anesthesia duration, opioid exposure, and intraoperative fluid balance interact to influence both recovery and PONV risk may optimize postoperative management. Therefore, this study aimed to identify perioperative predictors of prolonged LOS (> 3 days) and PONV after MBS, and focused on modifiable factors and the influence of surgical procedure type to guide anesthesia and recovery protocols.

## Methods

### Study design and patient selection

**Fig. 1:**
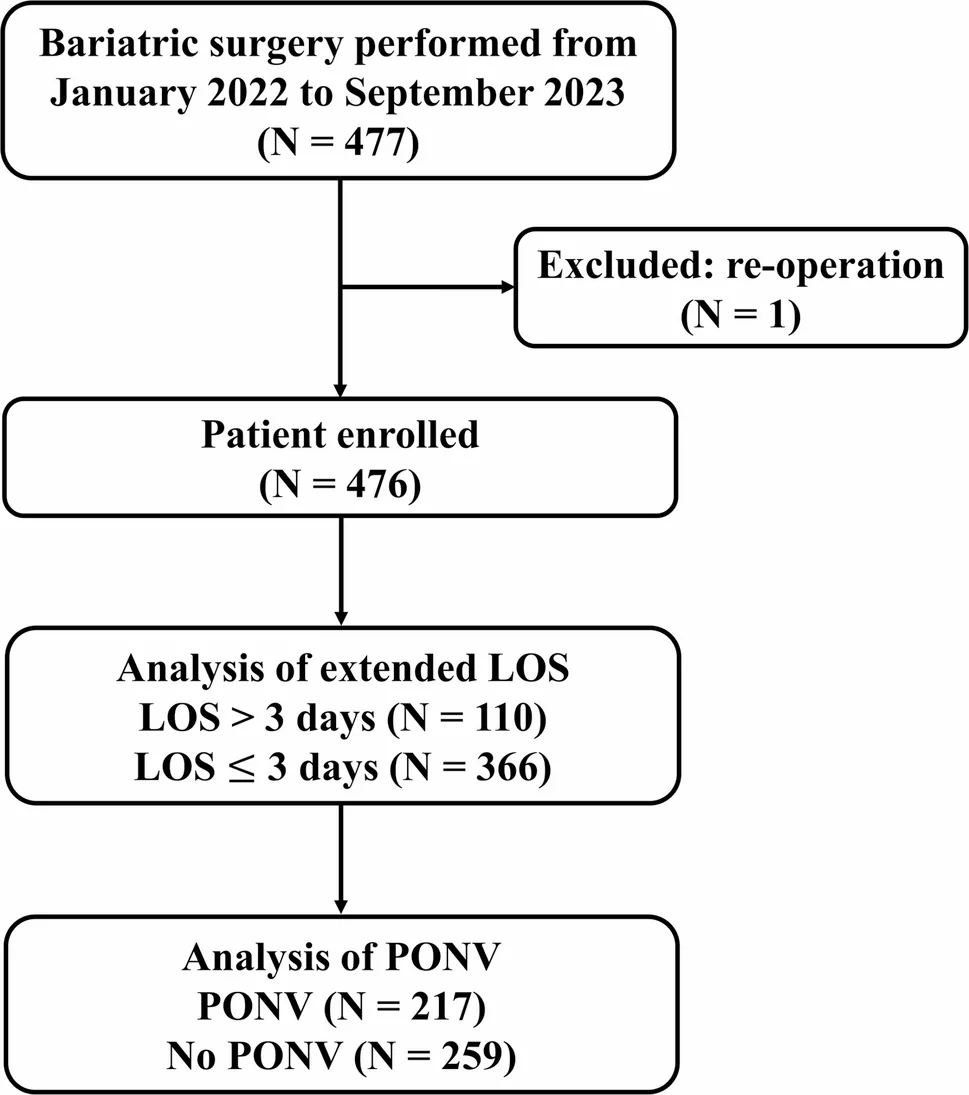
Flow diagram of patient selection and analysis process. A total of 477 patients undergoing bariatric surgery were screened; one patient was excluded due to emergency reoperation, leaving 476 for final analysis (*N* = 476 included). Abbreviations: LOS, length of stay; PONV, postoperative nausea and vomiting

This study was approved by the Institutional Review Board (IRB No. 202500075B0), and is reported in accordance with the STROBE guidelines for observational studies. Given its retrospective design and use of de-identified data, the requirement for informed consent was waived. Medical records of MBS patients from January 1, 2022, to September 30, 2023, were reviewed. A total of 477 patients were initially screened, and one was excluded due to an emergency reoperation within one day after surgery, and thus yielded a final cohort of 476 patients (Fig. 1). Baseline data collected included demographic characteristics (age, sex, height, weight, and BMI), American Society of Anesthesiologists (ASA) physical status, and associated medical problems such as hypertension, diabetes mellitus, and asthma (Table 1).

**Table 1:** Baseline characteristics of patients undergoing bariatric surgery

Variables	*N* (%) or median (IQR)	LOS ≤ 3 days (*N* = 366)	LOS > 3 days (*N* = 110)	*p* value	
Gender					
Female	287 (60.3%)	226 (61.7%)	61 (55.5%)		0.237
Male	189 (39.7%)	140 (38.3%)	49 (44.5%)		
Age (years)	43.0 (35.0–50.0)	43.0 (35.0–50.0)	44.0 (36.8–50.3)		0.381
Body height (cm)	164.0 (159.0–171.0)	164.0 (158.0–171.0)	165.0 (159.8–173.0)		0.329
Body weight (kg)	90.0 (75.0–108.0)	89.0 (75.0–106.0)	93.0 (75.8–117.3)		0.158
BMI (kg/m²)	33.37 (28.82–38.06)	33.30 (28.68–37.65)	33.93 (29.32–39.92)		0.179
ASA Classification					
I & II	379 (79.6%)	297 (81.1%)	82 (74.5%)		0.132
III	97 (20.4%)	69 (18.9%)	28 (25.5%)		
Hypertension	103 (21.6%)	70 (19.1%)	33 (30.0%)		0.015
Diabetes mellitus	86 (18.1%)	63 (17.2%)	23 (20.9%)		0.377
Asthma	21 (4.4%)	11 (3.0%)	10 (9.1%)		0.006
Type of surgery					
LAGB	253 (53.2%)	205 (56.0%)	48 (43.6%)		
LRYGB	223 (46.8%)	161 (44.0%)	62 (56.4%)		0.023
Anesthesia duration (hrs)	1.75 (1.50–2.00)	1.67 (1.50–2.00)	1.83 (1.67–2.27)		< 0.001
IV fluid infusion (mL/kg/hr)	2.89 (2.35–3.57)	2.91 (2.31–3.58)	2.80 (2.39–3.58)		0.955
Fentanyl dosage (mcg/kg/hr)	0.69 (0.51–0.88)	0.70 (0.55–0.93)	0.60 (0.44–0.82)		0.001
Sugammadex reversal	161 (33.8%)	118 (32.2%)	43 (39.1%)		0.183
Dexamethasone use	62 (13.0%)	45 (12.3%)	17 (15.5%)		0.388
PONV	217 (45.6%)	152 (41.5%)	65 (59.1%)		0.001

Values are presented as N (%) or median (IQR). Statistical tests: Kolmogorov–Smirnov, Mann–Whitney *U*, chi-square, or Fisher’s exact test, as appropriate. Statistical significance was set at *p* < 0.05

*Abbreviations*: *ASA* American Society of Anesthesiologists, *BMI* Body mass index, *IQR* Interquartile range, *IV* Intravenous, *LAGB* Laparoscopic adjustable gastric banding, *LOS* Length of stay, *LRYGB* Laparoscopic Roux-en-Y gastric bypass, *PONV* Postoperative nausea and vomiting

### Perioperative care pathway

All patients underwent a standardized preoperative evaluation and structured health education program. The study encompassed analyses of the most commonly performed operations included LAGB and LRYGB. LAGB is classified as a minimally invasive, purely gastric volume-limiting procedure, whereas LRYGB combines gastric volume limitation with intestinal bypass and is generally considered more complex [9]. All procedures were conducted by the same experienced bariatric surgeon. No patient in the analyzed cohort underwent concurrent hiatal hernia repair or adhesiolysis, and all procedures were primary (non-revisional) bariatric operations. During surgery, patients were placed in the reverse Trendelenburg position, and pneumoperitoneum was established with carbon dioxide insufflation maintained at below 15 mmHg under general anesthesia.

Anesthesia was induced using rapid sequence induction following preoxygenation, with intravenous fentanyl (1–2 mcg/kg ideal body weight [IBW]), propofol (1.5–2 mg/kg actual body weight or 2.3–3.5 mg/kg lean body weight), and a neuromuscular blocking agent, either rocuronium (0.6–1.2 mg/kg IBW) or cisatracurium (0.1–0.15 mg/kg IBW) [17]. Endotracheal intubation was performed orally, and mechanical ventilation was instituted. Anesthesia was maintained with sevoflurane inhalation (0.8–1.2 minimum alveolar concentration) and intermittent boluses of fentanyl (0.5–2 mcg/kg). Fluid administration, fentanyl prescription, and intraoperative parameters were closely monitored and recorded. Dexamethasone (5–10 mg, intravenous) was routinely administered as an antiemetic for PONV prophylaxis immediately following induction of anesthesia. No patient in the analyzed cohort received postoperative dexamethasone as rescue treatment; the dexamethasone variable in this analysis therefore reflects scheduled prophylactic administration only. Prochlorperazine, metoclopramide, or droperidol was given as rescue therapy if PONV occurred. After surgery, neuromuscular blockade was reversed with sugammadex (2–4 mg/kg) for rocuronium antagonism, or neostigmine (0.03–0.07 mg/kg) combined with atropine for cisatracurium blockage. For treating postoperative breakthrough pain, intravenous morphine (2–5 mg) and/or NSAID (such as ketorolac) would be administered as needed.

### Primary outcomes and predictor variables

At our institution, the standard clinical pathway for MBS involves a routine postoperative hospitalization of three days. The primary outcome was the postoperative LOS, defined as the number of days from the operation date to discharge, with the day of surgery designated as postoperative day 0. Based on institutional benchmarks, an extended LOS was defined as hospitalization longer than three days (LOS > 3 days), corresponding to the upper quartile of the cohort’s LOS distribution. This threshold reflects a deviation from the expected recovery trajectory within our standard postoperative protocol. The 3-day target is consistent with both the ERAS Society guidelines for bariatric surgery [25] and the median LOS reported in tertiary ERAS-managed bariatric cohorts [12]. Asian-Pacific MBS centers, including ours, characteristically report longer median LOS than North American or Western European centers, reflecting differences in patient phenotype and discharge practices, as documented in the most recent IFSO Global Registry Report [26]. The threshold was clinically anchored and prespecified before data analysis; we therefore did not perform a post hoc sensitivity analysis at a percentile-based cutoff, which would have addressed a different research question. Medical charts were reviewed to determine the causes of prolonged stay, commonly including delayed ambulation, insufficient pain control, persistent nausea or vomiting, or delayed drain removal. Discharge criteria were standardized and required stable vital signs, adequate pain control (visual analog scale < 4 at rest), tolerance of oral intake without nausea or vomiting, independent ambulation within the ward, and removal of intra-abdominal surgical drains.

To identify factors associated with prolonged hospitalization, univariable and multivariable logistic regression analyses were conducted with LOS > 3 days coded as 1 and ≤ 3 days as 0 (Table 2). Predictor variables included demographic characteristics (age, gender, BMI, ASA physical status, and type of surgery), intraoperative factors (anesthesia duration, intraoperative fentanyl dosage, and intravenous fluid administration rate), and associated medical problems (hypertension, diabetes mellitus, and asthma). Additional variables such as PONV, prophylactic dexamethasone administration, and use of sugammadex for neuromuscular reversal were also examined (Tables 2 and 3).

**Table 2:** Univariate analysis and multivariate logistic regression analysis for predictors of prolonged length of hospital stay (> 3 days) following bariatric surgery

Variables	*N* (%) or median (IQR)	Univariate analysis		Multivariable analysis	
		OR (95% CI)	*p* value	OR (95% CI)	*p value*
Gender					
Female	287 (60.3%)	1.00 (Reference)		1.00 (Reference)	
Male	189 (39.7%)	1.30 (0.84–2.00)	0.237	1.40 (0.84–2.35)	0.200
Age (years)	43.0 (35.0–50.0)	1.01 (0.99–1.03)	0.507	1.01 (0.99–1.04)	0.447
BMI (kg/m²)	33.37 (28.82–38.06)	1.02 (0.99–1.05)	0.145	1.02 (0.96–1.05)	0.835
Hypertension	103 (21.6%)	1.81 (1.12–2.94)	0.016	1.52 (0.82–2.82)	0.182
Diabetes mellitus	86 (18.1%)	1.27 (0.75–2.17)	0.378	0.75 (0.40–1.42)	0.379
Asthma	21 (4.4%)	3.23 (1.33–7.82)		2.99 (1.09–8.18)	0.033
Type of surgery					
LAGB	253 (53.2%)	1.00 (Reference)	0.009	1.00 (Reference)	
LRYGB	223 (46.8%)	1.65 (1.07–2.53)	0.023	1.65 (0.94–2.87)	0.079
Anesthesia duration (hrs)	1.75 (1.50–2.00)	2.83 (1.84–4.36)	< 0.001	3.05 (1.70–5.50)	< 0.001
IV fluid infusion (mL/kg/hr)	2.89 (2.35–3.57)	1.11 (0.85–1.30)	0.199	1.41 (1.11–1.78)	0.005
Fentanyl dosage (mcg/kg/hr)	0.69 (0.51–0.88)	0.47 (0.22–0.99)	0.048	1.10 (0.34–3.57)	0.876
Sugammadex reversal	161 (33.8%)	1.35 (0.87–2.10)	0.184	1.32 (0.80–2.19)	0.281
Dexamethasone use	62 (13.0%)	1.30 (0.71–2.39)	0.389	0.95 (0.47–1.89)	0.875
PONV	217 (45.6%)	2.03 (1.32–3.14)	0.001	2.09 (1.29–3.40)	0.003

*Abbreviations*: *BMI* Body mass index, *CI* Confidence interval, *IQR* Interquartile range, *IV* Intravenous, *LAGB* Laparoscopic adjustable gastric banding, *LRYGB* Laparoscopic Roux-en-Y gastric bypass, *OR* Odds ratio, *PONV* Postoperative nausea and vomiting

**Table 3:** Comparison of demographic and perioperative variables according to postoperative nausea and vomiting status

Variables	*N* (%) or median (IQR)	No PONV (*N* = 259)	PONV (*N* = 217)	*p* value
Gender				
Female	287 (60.3%)	136 (52.5%)	151 (69.6%)	< 0.001
Male	189 (39.7%)	123 (47.5%)	66 (30.4%)	
Age (years)	43.0 (35.0–50.0)	44.0 (36.0–52.0)	42.0 (34.0–48.0)	0.040
Body height (cm)	164.0 (159.0–171.0)	165.0 (159.0–173.0)	163.0 (158.0–170.0)	0.029
Body weight (kg)	90.0 (75.0–108.0)	87.0 (75.0–108.0)	90.0 (75.0–108.0)	0.853
BMI (kg/m²)	33.37 (28.82–38.06)	33.23 (27.91–37.83)	33.59 (29.35–38.38)	0.307
ASA classification				
I & II	379 (79.6%)	207 (79.9%)	172 (79.3%)	0.277
III	97 (20.4%)	52 (20.1%)	45 (20.7%)	
Hypertension	103 (21.6%)	66 (25.5%)	37 (17.1%)	0.026
Diabetes mellitus	86 (18.1%)	45 (17.4%)	41 (18.9%)	0.668
Asthma	21 (4.4%)	13 (5.0%)	8 (3.7%)	0.481
Type of surgery				
LAGB	253 (53.2%)	153 (59.1%)	100 (46.1%)	0.005
LRYGB	223 (46.8%)	106 (40.9%)	117 (53.9%)	
Anesthesia duration (hrs)	1.75 (1.50–2.00)	1.67 (1.42–2.00)	1.75 (1.58–2.08)	0.010
IV fluid infusion (mL/kg/hr)	2.89 (2.35–3.57)	2.86 (2.33–3.64)	2.95 (2.38–3.49)	0.925
Fentanyl dosage (mcg/kg/hr)	0.69 (0.51–0.88)	0.71 (0.55–0.91)	0.66 (0.49–0.84)	0.077
Sugammadex reversal	161 (33.8%)	85 (32.8%)	76 (35.0%)	0.613
Dexamethasone use	62 (13.0%)	23 (8.9%)	39 (18.0%)	0.003
LOS (days)	3.0 (2.0–3.0)	3.0 (2.0–3.0)	3.0 (2.0–4.0)	< 0.001

Values are presented as N (%) or median (IQR). Statistical tests: Kolmogorov-Smirnov, Mann-Whitney *U*, chi-square, or Fisher’s exact test, as appropriate. Statistical significance was set at *p* < 0.05. Comparisons in this table are exploratory and intended to characterize the cohort; *p* values are not adjusted for multiple comparisons and should not be interpreted as confirmatory

*Abbreviations*: *ASA* American Society of Anesthesiologists, *BMI* Body mass index, *IQR* Interquartile range, *IV* Intravenous, *LAGB* Laparoscopic adjustable gastric banding, *LOS* Length of stay, *LRYGB* Laparoscopic Roux-en-Y gastric bypass, *PONV* Postoperative nausea and vomiting

The secondary outcome was PONV, defined as any documented nausea, retching, or vomiting within 48 h after surgery. Patients were classified as having PONV (1) or no PONV (0). Group comparisons for PONV were performed using univariable analyses (Table 3). Covariates included gender, age, BMI, anesthesia duration, intraoperative intravenous fluids, fentanyl dosage, ASA classification, hypertension, diabetes, asthma, type of surgery, sugammadex reversal, and dexamethasone as prophylactic antiemetics use.

### Statistical analysis

**Fig. 2:**
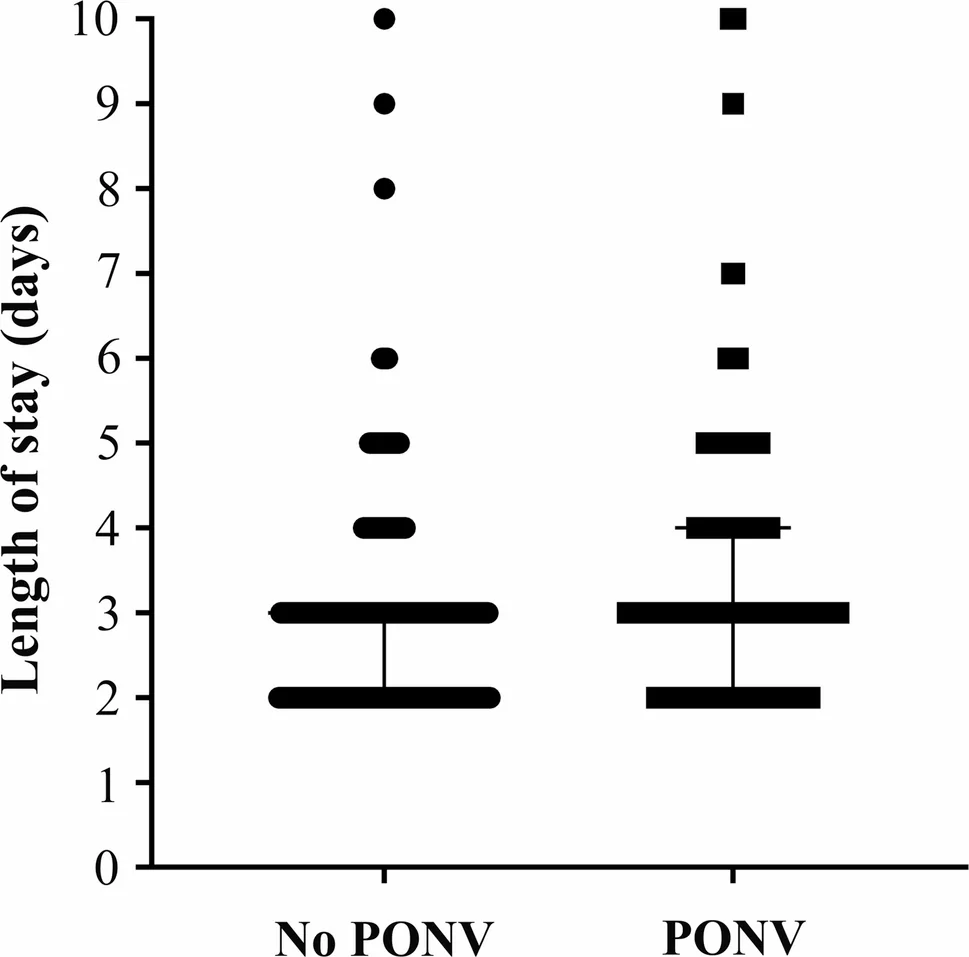
Comparison of postoperative length of stay (LOS) between patients with and without postoperative nausea and vomiting (PONV). The figure displays box plots with individual data points. The central line indicates the median, and the box represents the interquartile range (IQR, 25th–75th percentile). Abbreviations: LOS, length of stay; PONV, postoperative nausea and vomiting

Continuous variables were expressed as median (interquartile range [IQR]) and categorical variables as N (%). Group comparisons used the Mann-Whitney *U* test for nonparametric data and the chi-square or Fisher’s exact test for categorical data. Prolonged LOS was defined as hospitalization > 3 days, which exceeds the routine discharge schedule for patients undergoing MBS. Univariate analyses were performed to identify preoperative and perioperative factors associated with prolonged LOS. Significant variables from the univariate analysis were subsequently entered into a stepwise multivariate logistic regression model to determine independent predictors (Table 2). Odds ratios (OR) with 95% confidence intervals (CI) were calculated, and statistical significance was defined as *p* < 0.05. Box plots illustrated the full distribution of hospital LOS stratified by with or without PONV (Fig. 2). Kernel-density envelopes with embedded boxplots display medians, interquartile ranges, and outliers. The plots enable visual comparison of dispersion and skewness between with or without PONV groups and facilitate assessment of PONV-associated differences in recovery duration and LOS variability. Analyses were performed using SPSS version 22.0 (IBM Corp., Armonk, NY, USA). Body mass index was modeled as a continuous variable in multivariable logistic regression to maximize statistical efficiency and avoid information loss inherent to categorization. Predictors entered into the multivariable model were prespecified on biological and clinical grounds rather than selected by data-driven procedures. The events-per-variable (EPV) ratio was 10 (110 events for 11 predictors), meeting the conventional Peduzzi threshold for stable maximum-likelihood estimation [27]. No complete or quasi-complete separation was observed; we therefore retained standard maximum-likelihood logistic regression rather than applying Firth penalization. Comparisons in Table 3 are descriptive and hypothesis-generating, and p-values are not adjusted for multiplicity.

## Results

### Patient characteristics and prolonged hospital stay

A total of 476 patients undergoing MBS were included in the final analysis, comprising 287 females (60.3%) with a median age of 43.0 years (IQR: 35.0–50.0). Baseline characteristics revealed a median BMI of 33.37 kg/m^2^ (IQR: 28.82–38.06), with 79.6% classified as American Society of Anesthesiologists (ASA) physical status I–II. Pre-existing comorbidities included hypertension (21.6%) and diabetes mellitus (18.1%). The median anesthesia duration was 1.75 h (IQR: 1.50–2.00), and the overall median hospital LOS was 3.0 days. PONV occurred in 45.6% of patients, and 23.1% (*N* = 110) experienced prolonged LOS (> 3 days). The cohort included 253 patients undergoing LAGB (53.2%) and 223 patients undergoing LRYGB (46.8%). There were no intraoperative complications in the analyzed cohort, and no patient required intraoperative blood transfusion or unplanned conversion. No major postoperative complications other than PONV (e.g., surgical site infection, staple-line leak, deep venous thrombosis) occurred. The single patient excluded from analysis—who experienced postoperative bleeding requiring emergency reoperation on postoperative day 1—was excluded a priori to preserve cohort homogeneity (Fig. 1).

Univariable analysis (Table 1) comparing patients with prolonged LOS to those with shorter stays (≤ 3 days) identified several statistically significant differences. The prolonged LOS group demonstrated higher prevalences of hypertension (30.0% vs. 19.1%, *p* = 0.015) and asthma (9.1% vs. 3.0%, *p* = 0.006), and required a significantly longer anesthesia duration (median 1.83 vs. 1.67 h, *p* < 0.001). Postoperatively, this group also experienced higher rates of PONV (59.1% vs. 41.5%, *p* = 0.001). Interestingly, the prolonged LOS group received a lower median intraoperative fentanyl dosage (0.60 vs. 0.70 mcg/kg/hr, *p* = 0.001). Patients undergoing LRYGB had a higher proportion in the prolonged LOS group (56.4%) than LAGB patients (43.6%), a statistically significant difference (*p* = 0.023) (Table 1).

### Factors associated with prolonged LOS

Out of 476 patients, 110 (23.1%) experienced a prolonged hospital stay beyond 3 days. In univariate comparisons (Table 2), patients with prolonged LOS had significantly higher rates of hypertension and asthma, LRYGB patients, longer anesthesia duration, and more frequent PONV than those discharged by postoperative day 3 (all* p *< 0.05). By contrast, baseline characteristics such as age, sex, BMI, and ASA physical status did not differ significantly between the groups.

Multivariate logistic regression was performed to determine independent predictors of extended LOS (Table 2). The final model identified four independent factors significantly associated with prolonged hospitalization. Longer anesthesia duration remained a highly significant predictor, and increased the odds of a prolonged stay by over three-fold (OR: 3.05, 95% CI 1.70–5.50;* p* < 0.001). Higher intraoperative intravenous fluid rate was also independently associated with prolonged LOS (OR: 1.41, 95% CI 1.11–1.78; *p* = 0.005). The occurrence of PONV conferred a two-fold increase in the risk of extended hospitalization (OR: 2.09, 95% CI 1.29–3.40; *p* = 0.003) (Table 2; Fig. 2). Furthermore, a patient history of asthma independently predicted prolonged LOS (OR: 2.99, 95% CI 1.09–8.18; *p* = 0.033).

Importantly, while the type of surgery (LRYGB vs. LAGB) showed a significant association in univariate analysis (*p* = 0.023), it was not identified as an independent predictor in the multivariable model (OR: 1.65, 95% CI 0.94–2.87; *p* = 0.079). This suggests that the impact of the more complex LRYGB procedure on LOS may be mediated or confounded by other independent variables, such as anesthesia duration and PONV incidence. Hypertension, which was significant in the univariate analysis, lost its statistical significance after adjustment for other variables (*p* = 0.182). Other factors, including age, sex, BMI, and ASA status, were not significant independent predictors in the adjusted model. These findings emphasize the combined impact of modifiable intraoperative management parameters (anesthesia time and fluid administration), postoperative complications (PONV), and intrinsic patient associated medical problems (asthma) on early recovery trajectories after MBS.

### Factors associated with PONV

In this cohort, 217 (45.6%) experienced PONV (Table 3). Compared with those without PONV (*N* = 259), affected patients were more often female (69.6% vs. 52.5%; *p* < 0.001), indicating a significant sex-related susceptibility. They were also slightly younger (median 42.0 vs. 44.0 years; *p* = 0.040) and shorter in stature (163.0 vs. 165.0 cm; *p* = 0.029), both showing statistically significant differences. Perioperatively, PONV was associated with longer anesthesia duration (1.75 vs. 1.67 h; *p* = 0.010), and thus underscored the relevance of operative/anesthetic time to emetogenic risk. Patients who developed PONV had a longer LOS overall (*p* < 0.001), supporting the clinical and logistical impact of this complication. Prophylactic antiemetic dexamethasone was administered more frequently in the PONV group (18.0% vs. 8.9%; *p* = 0.003); this likely reflects risk-based or reactive prescribing patterns rather than a protective effect and should be interpreted accordingly. In contrast, hypertension was less prevalent among patients with PONV (17.1% vs. 25.5%; *p* = 0.026). No significant differences were observed for BMI, diabetes mellitus, asthma, ASA classification, intraoperative fentanyl dose, intravenous fluid rate, sugammadex use, or other baseline characteristics. Collectively, these data emphasize female sex, younger age, shorter height, and longer anesthesia duration as significant correlates of PONV, with measurable consequences for postoperative recovery.

## Discussion

This study identified several perioperative factors significantly associated with prolonged LOS and PONV following MBS. In a cohort of 476 patients managed under a standardized pathway, longer anesthesia duration, greater intraoperative intravenous fluid administration, the occurrence of PONV, and a history of asthma were independent predictors of extended LOS (Table 1). These findings align with the broader context in which obesity is tightly linked to cardiometabolic disease and reduced survival [6, 28], while MBS improves associated medical problems, reduces medication burden, and confers survival benefits [6–8]. Optimizing perioperative care within Enhanced Recovery After Surgery (ERAS) frameworks therefore remains central to improving outcomes and reducing complications [25].

### Surgical procedure type and LOS

Analysis comparing surgical procedure type (LRYGB vs. LAGB, with LAGB as the reference group) showed differential effects on recovery metrics. Univariate analysis indicated LRYGB was significantly associated with prolonged LOS (OR: 1.65, *p* = 0.023). However, this association lost statistical significance in the multivariate regression model (OR: 1.65, 95% CI 0.94–2.87; *p* = 0.079). This suggests the LRYGB effect is mediated by other factors, primarily its greater technical complexity, which leads to longer anesthesia duration and higher physiological stress compared to the LAGB procedure.

Furthermore, LRYGB was significantly linked to a higher incidence of postoperative nausea and vomiting (PONV; *p* = 0.005). Since PONV itself is a strong independent predictor of prolonged LOS (OR: 2.09), the loss of surgical type independence underscores that the extended stay is driven by longer operative time and the subsequent risk of complications like PONV, rather than the procedure type alone. These findings necessitate tailored ERAS protocols, allocating greater resources to LRYGB patients. By including PONV in the multivariable model, we estimated the direct effect of procedure type net of its mediation through PONV; the apparent attenuation should not be read as evidence against a procedure-type effect on LOS but rather as quantitative evidence that the procedure-type effect operates substantially via the PONV pathway. The clinical implication is that aggressive procedure-specific multimodal PONV prophylaxis is particularly important for LRYGB patients. A formal causal mediation analysis was not performed because the sequential ignorability assumption required for unbiased decomposition of total, direct, and indirect effects cannot be guaranteed in a retrospective chart-abstracted dataset; this remains an important question for prospective work.

### Anesthesia Duration and LOS

Prolonged anesthesia/operative time was an independent predictor of extended LOS in our cohort, with an odds ratio of 3.05 (*p* < 0.001) (Table 2), and aligns with large database analyses of sleeve gastrectomy, which identify longer operative time as an independent risk factor for > 3-day hospitalization [13]. Mechanistically, extended exposure to general anesthetics in patients with obesity can impair early recovery by increasing residual sedation, reducing ventilatory responsiveness, and delaying mobilization, thereby predisposing to hypoxemia, atelectasis, and functional setbacks [29, 30]. Procedurally, longer operations may also signal greater technical complexity, such as adhesions, difficult dissection, or concomitant repairs. In particular, concurrent hiatal hernia repair during sleeve gastrectomy is associated with significantly longer operative time, consistent with added steps such as crural approximation and mesh or suture reinforcement, which inherently prolong anesthesia and can lengthen postoperative convalescence [31]. Optimizing surgical efficiency and standardizing intraoperative workflows are therefore critical programmatic goals. Anesthesia duration in this cohort should be regarded as a composite surrogate marker of procedural and patient-related complexity rather than a directly modifiable target. The clinical implication is therefore not that anesthesia time should be artificially shortened, but that patients with intraoperatively prolonged operations should be flagged at the end of surgery for intensified postoperative monitoring, proactive multimodal antiemetic coverage, and earlier mobilization planning. An exploratory dose–response analysis reinforced this interpretation: when anesthesia duration was categorized into quartiles (Q1, 0.50–1.50 h; Q2, 1.50–1.75 h; Q3, 1.75–2.00 h; Q4, 2.00–5.25 h), the unadjusted proportion of prolonged LOS rose monotonically from 12.9% in Q1 to 22.1%, 25.7%, and 35.2% in Q2–Q4, respectively (Q4 vs. Q1: OR 3.69, 95% CI 1.95–6.97; P for trend < 0.001; Supplemental Table S2). This clear gradient contrasts with the absence of a dose–response for intraoperative fluid rate (P for trend = 0.923; Supplemental Table S1), further supporting the distinction between anesthesia duration as a genuine complexity marker and fluid rate as a confounding-by-indication signal. Whether targeted process interventions (e.g., parallel-processing pre-induction workflows or structured intraoperative handover) translate into measurably shorter LOS will require prospective evaluation.

### Fluid management and LOS

Intraoperative fluid administration emerged as an independent determinant of LOS (Table 2). Excess fluid can exacerbate intestinal edema, delay gastrointestinal recovery, and increase complications; conversely, hypovolemia may precipitate hypotension and organ hypoperfusion [32, 33]. The analysis showed that a higher intravenous fluid rate increased the odds of prolonged stay (OR: 1.41, *p* = 0.005) (Table 2). This observation reinforces the principle of judicious fluid stewardship during major surgery. This observation aligns with prior evidence in major abdominal surgery supporting goal-directed fluid therapy to optimize stroke volume while avoiding overload [15], and is concordant with reports linking higher early postoperative fluid exposure to longer hospitalization in bariatric patients [12]. While randomized data specific to fluid targets in MBS remain limited, RCTs combining total intravenous anesthesia with goal-directed fluid therapy reduce PONV in MBS, and meta-analyses in major surgery support hemodynamic-guided fluids to avoid both under- and over-resuscitation [15]. The association between higher intraoperative fluid rate and prolonged LOS should be interpreted with caution and is best understood as confounding by indication: patients receiving higher fluid rates were almost certainly those with greater intraoperative hemodynamic variability, transient hypotension, or vasopressor requirement, in which case the elevated fluid rate is a downstream marker of instability rather than the proximate driver of delayed recovery. Fine-grained intraoperative hemodynamic data (continuous blood pressure trajectories, vasopressor dosing) were not retrievable from the chart-abstracted dataset and could not be modeled. In an exploratory sensitivity analysis, intraoperative fluid rate was categorized into quartiles (Q1, 1.04–2.35; Q2, 2.35–2.88; Q3, 2.89–3.57; Q4, 3.57–15.62 mL/kg/hr; *N* = 119 per quartile); no significant unadjusted dose–response trend was observed for either prolonged LOS (Q2 vs. Q1: OR 1.74, 95% CI 0.95–3.18; Q3: OR 1.11, 95% CI 0.59–2.09; Q4: OR 1.22, 95% CI 0.66–2.29; P for trend = 0.923) or PONV (P for trend = 0.902). The absence of a monotonic gradient in the unadjusted categorical analysis, contrasted with the significant adjusted continuous association (OR 1.41, *P* = 0.005), is consistent with the confounding-by-indication interpretation and suggests that the adjusted signal reflects residual confounding by unmeasured hemodynamic instability rather than a simple volume–outcome gradient.

### PONV and recovery

PONV was a frequent complication, affecting 45.6% of the cohort, and demonstrated a highly significant independent association with extended LOS (OR: 2.09, *p* = 0.003) (Tables 2 and 3, and Fig. 2). Patients experiencing PONV were approximately twice as likely to remain hospitalized longer than three days, and thus incurred its substantial clinical and economic burden. The clinical consequences often manifest as delayed oral intake, dehydration, and functional setbacks, potentially leading to unplanned readmission. Prior work also links postoperative nausea with longer hospitalization and higher resource use [34]. Multiple established risk factors, such as female sex, younger age, volatile anesthetics, and postoperative opioids/patient-controlled analgesia, are consistently associated with higher PONV risk [18, 20, 35]. The biological basis for the female preponderance remains incompletely understood; fluctuations in estrogen and progesterone across the menstrual cycle may modulate central serotonergic and dopaminergic pathways that govern emetogenic susceptibility, yet menstrual cycle phase and menopausal status were not recorded in our dataset and warrant prospective investigation. Although standardized prophylaxis was employed, the residual PONV suggests that conventional “one-size-fits-all” regimens may be inadequate for high-risk individuals [20, 34]. Guidelines endorse opioid-sparing and multimodal antiemetic strategies. Randomized and observational bariatric data suggest benefits for recovery, with mixed but generally favorable signals for PONV [18, 19]. Our results support a risk-stratified, multimodal antiemetic strategy that minimizes emetogenic exposures (volatile anesthetics and opioids) and combines agents from different classes for high-risk patients (Table 3) [16, 21]. The paradoxically lower intraoperative fentanyl dosing observed in prolonged-stay patients may reflect conservative opioid use in more complex or higher-risk cases, with subsequent reliance on postoperative analgesia. This pattern underscores the need for balanced, opioid-sparing strategies that still ensure adequate intraoperative analgesia. PONV frequently precipitates delayed discharge or unplanned readmission, often via poor oral tolerance and dehydration [36]. The higher prevalence of dexamethasone use in the PONV group is most likely confounding by indication: clinicians preferentially administered prophylaxis to patients perceived as having higher baseline emetogenic risk—typically female, younger, non-smokers, with anticipated higher postoperative opioid exposure—the very phenotype most likely to develop PONV regardless of prophylaxis. Causal antiemetic efficacy cannot therefore be inferred from this observational pattern. PONV after laparoscopic bariatric surgery is reported at incidences as high as 60–80% in the absence of comprehensive prophylaxis [37] and single-agent prophylaxis has consistently proven insufficient in this high-risk population, supporting multimodal antiemetic pathways combining at least two pharmacologic agents with non-pharmacologic strategies.

### Asthma and postoperative course

Asthma independently predicted prolonged LOS, with a substantial odds ratio of 2.99 (*p* = 0.033) in our analysis (Table 2). In patients with severe obesity, reduced functional residual capacity with greater airway closure leads to higher airway resistance and ventilation–perfusion mismatch. Mechanical ventilation can amplify pro-inflammatory responses (ventilator-induced lung injury), and predispose to hypoxemia and delayed recovery [38, 39]. Although MBS is associated with reduced asthma medication requirements over time, the perioperative window remains vulnerable and warrants targeted respiratory optimization [40]. Practical measures include preoperative inhaled bronchodilator optimization, intraoperative lung-protective ventilation with low driving pressures and recruitment as appropriate, and early mobilization with incentive spirometry [25, 41].

### Non-independent demographic factors

In our cohort, hypertension was significant in univariate but not multivariable analyses (Tables 1 and 2), suggesting confounding by age or ASA status rather than a direct effect on LOS. Similarly, sex, BMI, and diabetes were not independent predictors after adjustment, aligning with reports that perioperative management decisions and procedure type and peri/postoperative complications (e.g., case logistics, routine postoperative studies) outweigh static demographics in shaping early recovery [14, 42]. Notably, hypertension has been associated with higher 30-day readmission risk in large registry studies, and shows the value of targeted post-discharge surveillance and timely outpatient follow-up to detect dehydration, uncontrolled pain, or PONV that may precipitate returns to care and signal early complications [43].

### Integrated Interpretation and Implications

A key contribution of this study is the concurrent evaluation of PONV and prolonged LOS within the same standardized bariatric cohort. These outcomes are interdependent: PONV can delay oral intake and hydration, leading to extended hospitalization, while prolonged stays may expose patients to environmental and pharmacologic factors that amplify nausea. Recognizing this bidirectional relationship supports integrated protocols that simultaneously address anesthetic duration, fluid stewardship, and emetogenic exposures. The finding that LRYGB was significant only in the univariate model, and losing predictive power once anesthesia duration and PONV were considered. These data suggest that the perceived delay associated with LRYGB is largely attributable to the increased time required for the surgery and the higher incidence of complications, not the procedure type as a singular, independent variable.

Implementing programmatic adherence to ERAS is paramount. This includes prioritizing efficiency to shorten anesthesia time, maintaining strict goal-directed fluid management to avoid overload, and utilizing aggressive, procedure-specific multimodal antiemetic prophylaxis (Tables 2 and 3) [19, 44]. Since LRYGB patients face higher risks in both procedural complexity and PONV incidence, clinical pathways should differentiate recovery expectations by procedure, establishing highly optimized, accelerated discharge protocols for LAGB patients while providing extended initial monitoring and more focused perioperative management for LRYGB patients. From a quality-improvement perspective, programmatic adherence to ERAS with routine audit and feedback can guide pathway adjustments; dashboards tracking anesthesia time, weight-adjusted fluid delivery, PONV rates, and asthma-specific respiratory metrics may support real-time optimization [25].

### Limitations

This retrospective single-center design limits causal inference and external generalizability [45]. We lacked granular markers of surgical complexity (e.g., hernia repair, hiatal dissection) and airway difficulty, which may confound anesthesia duration. Although discharge criteria were standardized, institutional nuances may still influence LOS. In particular, non-clinical factors such as weekend discharge logistics, social-support availability, and administrative variation in discharge coordination may modulate LOS independently of clinical readiness; granular data on these system-level determinants were not retrievable from the present dataset. PONV ascertainment relied on clinical documentation and may undercount mild episodes. Finally, while our models adjusted for key covariates, unmeasured confounding cannot be excluded. In particular, data on obstructive sleep apnea status were not routinely captured in the anesthesia record and could not be included as a covariate; given the well-established association between obstructive sleep apnea and perioperative respiratory complications in patients with obesity, this represents a potentially important unmeasured confounder. Similarly, comorbidities were recorded as binary variables, and we could not stratify by disease severity (e.g., insulin-dependent versus oral-agent-controlled diabetes, number of antihypertensive agents, or inhaler regimen complexity for asthma), which may have obscured dose–response relationships between comorbidity burden and prolonged LOS. Nevertheless, the relatively large sample, uniform pathway, and comprehensive covariate set enhance the credibility and clinical utility of our findings. Several additional caveats merit comment. The cohort’s lower median BMI of 33.4 kg/m² reflects regionally appropriate Asian indications for metabolic surgery, in which surgery is offered at lower BMI thresholds owing to greater visceral adiposity and a higher prevalence of cardiometabolic comorbidity [26]; the high proportion of LAGB (53.2%) reflects practice patterns at our institution during the study period and may limit generalizability to centers in which sleeve gastrectomy currently predominates. The events-per-variable ratio of the multivariable logistic regression was at the conventional threshold of 10, and although our predictor set was prespecified rather than data-driven and no separation was observed, model coefficients should be interpreted as estimates that may benefit from external validation in a larger or multicenter cohort.

## Conclusion

Prolonged anesthesia duration, greater intraoperative fluid administration, PONV, and asthma independently contributed to extended hospital stay after MBS. The procedural difference between LRYGB and LAGB was significantly associated with prolonged LOS only in unadjusted analysis, and highlighted the role of modifiable intraoperative and postoperative factors in driving recovery outcomes. Programs should prioritize workflow efficiency, goal-directed fluid therapy, risk-stratified multimodal antiemetic prophylaxis, and proactive asthma management within ERAS. Future prospective studies integrating hemodynamic-guided fluids and opioid-sparing techniques, with concurrent evaluation of PONV and LOS as co-primary endpoints, are warranted to validate these associations and refine evidence-based pathways for MBS [19, 24, 46].

## Supplementary Information

Supplementary Material 1.

Supplementary Material 2.

Supplementary Material 3.

## Data Availability

The datasets used during the current study are available from the corresponding author on reasonable request.

## Abbreviations

ASA: American Society of Anesthesiologists
BMI: Body mass index
CI: Confidence interval
ERAS: Enhanced recovery after surgery
IBW: Ideal body weight
IRB: Institutional Review Board
IQR: Interquartile range
IV: Intravenous
LAGB: Laparoscopic adjustable gastric banding
LOS: Length of stay
LRYGB: Laparoscopic Roux-en-Y gastric bypass
MBS: Metabolic bariatric surgery
NSAID: Nonsteroidal anti-inflammatory drug
OR: Odds ratio
PONV: Postoperative nausea and vomiting
RCT: Randomized controlled trial
SPSS: Statistical Package for the Social Sciences
STROBE: Strengthening the Reporting of Observational Studies in Epidemiology
